# Genetic Relatedness, Emotional Closeness and Physical Aggression: A Comparison of Full and Half Sibling Experiences

**DOI:** 10.5964/ejop.v16i1.1620

**Published:** 2020-03-03

**Authors:** Roxanne Khan, Gayle Brewer, John Archer

**Affiliations:** a School of Psychology, University of Central Lancashire, Preston, United Kingdom; b School of Psychology, University of Liverpool, Liverpool, United Kingdom; Department of Psychology and Counselling, Webster University Geneva, Geneva, Switzerland

**Keywords:** family violence, conflict, Hamilton’s rule, sibling violence, weapon use

## Abstract

Two studies investigated whether perceived closeness of siblings, and aggression between siblings, were associated with genetic relatedness. In following Hamilton’s rule, we predicted that as the coefficient of relatedness between siblings increased, emotional closeness would also increase while conflict would decrease. Contrary to the predictions, we found no effect of genetic relatedness in Study 1 when we compared participants’ (n = 240) ratings of emotional closeness; participants also reported significantly higher levels of conflict with full siblings than with half siblings. In Study 2, participants (n = 214) also reported a higher frequency of physical aggression with full siblings than with half siblings. These findings were contrary to the prediction from Hamilton’s rule. We discuss them in relation to parental investment in biological and non-biological offspring.

According to “Hamilton’s Rule” (Hamilton, 1964a, 1964b), genetic relatedness predicts the form and frequency of altruistic and competitive behavior, such that people are most altruistic and least competitive with those to whom they are most closely related. Within the familial environment, research has consistently documented the disparate treatment of genetically unrelated children by stepparents compared with their own biological offspring. In particular, stepchildren are at a far greater risk of neglect, physical maltreatment, and infanticide, than genetically related children (e.g., Daly & Wilson, 1994, 2008; Harris, Hilton, Rice, & Eke, 2007; Tooley, Karakis, Stokes, & Ozanne-Smith, 2006; Weekes-Shackelford & Shackelford, 2004; Wilson, Daly, & Weghorst, 1980). The present studies explore the influence of genetic relatedness on closeness and aggression between siblings. Genetic relatedness can be expressed by the coefficient of relatedness (*r*), from Wright (1922), which denotes the likelihood that two people have the same gene as a result of common inheritance. From Hamilton’s Rule, we should expect people to report greater emotional closeness and less intentional aggression towards full (*r* = .5) than half siblings (*r* = .25).

## Emotional Closeness

Previous research indicates that genetic relatedness predicts emotional closeness and social support (Neyer & Lang, 2003). For example, coalitions are more frequently formed with those who are closely related (Dunbar, Clark, & Hurst, 1995) and people are more likely to incur costs for kin than non-kin (Burnstein, Crandall, & Kitayama, 1994). The importance of genetic relatedness appears to extend to sibling relationships. Fraley and Tancredy’s (2012) exploration of national data on 28,169 sibling relationships showed that twins were more likely to be attached to their siblings than non-twin siblings and identical (monozygotic) twins were more likely than fraternal (dizygotic) twins to be strongly attached to one another. These findings are consistent with previous research that demonstrates that (1) non-twins are less positive towards each other than identical or fraternal twins, with unrelated siblings least positive of all (Reiss, Neiderhiser, Hetherington, & Plomin, 2000); (2) there is more severe grief following the death of a twin than a non-twin sibling (Segal, Wilson, Bouchard, & Gitlin, 1995); (3) there is greater cooperation between identical than fraternal twins (Segal, 2005); and (4) there is greater contact between full than half siblings (Pollet, 2007; Tanskanen & Danielsbacka, 2014; White & Riedmann, 1992).

Furthermore, differences between full and half sibling interactions were found in a polygynous Mormon community (Jankowiak & Diderich, 2000), where half siblings share a father and have different mothers but are reared in the same household as full siblings. Despite attempts to reduce the significance of genetic relatedness in this community, in the interests of social cohesion, individuals reported greater affection for full than half siblings (Jankowiak & Diderich, 2000). These findings are consistent with the assertion that humans should have developed the ability to distinguish full from half siblings and to act more cooperatively with full siblings (Buss, 1999; Daly, Salmon, & Wilson, 1997). However, other studies report that genetically related siblings (full and half) were both more positive *and* more negative in their relationship than were unrelated siblings (Hetherington et al., 1999; Reiss et al., 1994). Thus we investigate sibling relatedness in the context of both closeness and conflict.

## Conflict and Aggression

While siblings provide a source of support in human families (Tucker, McHale, & Crouter, 2001), they also represent competition for valued resources, and this is apparent throughout the animal kingdom (Mock & Parker, 1997). As the number of children in the household increases, there is a decline in the amount of parental time and investment allocated to each child (Black, Devereux, & Salvanes, 2005). More generally, larger family size is associated with lower offspring fitness in other animals (Lack, 1947; Roff, 2002). The number of siblings within the household is particularly detrimental to later-born children (Lawson & Mace, 2009). For example, children with additional older siblings have a lower height and growth rate (Lawson & Mace, 2008) and a lower level of educational attainment and wealth (Steelman, Powell, Werum, & Carter, 2002). Consistent with the importance of household resources, short birth intervals (increasing the intensity of resource competition) are associated with a greater risk of child mortality (Whitworth & Stephenson, 2002) and sibling conflict often centers on personal possessions (Felson, 1983; McGuire, Manke, Eftekhari, & Dunn, 2000; Raffaelli, 1992). Competition for resources may be particularly intense within middle income or poorer households where parents may focus their investment on a few of their children, creating substantial inequalities amongst siblings (Dahan & Gaviria, 2003).

Aggressive altercations among siblings are commonplace, widespread, and occur in different cultures (Rapoza, Cook, Zaveri, & Malley-Morrison, 2010; Relva, Fernandes, & Costa, 2013), although it is often minimized (Khan & Rogers, 2015), not only by victims but also by family members and professionals (Khan & Brewer, in press; McDonald & Martinez, 2016; Phillips, Phillips, Grupp, & Trigg, 2009). Indeed, physical aggression is often viewed as a normal part of the sibling relationship (Caspi, 2012; Hardy, 2001; Kettrey & Emery, 2006), despite the negative emotional and behavioral consequences of this behavior (Caffaro & Conn-Caffaro, 1998; Kashani, Daniel, Dandoy, & Holcomb, 1992). The widespread acceptance of physical aggression against siblings is further reflected in the absence of laws to protect victims from this maltreatment (Stock, 1993). Hence, although sibling aggression is likely to be the most prevalent form of family violence (e.g., Eriksen & Jensen, 2006, 2009; Finkelhor, Turner, & Ormrod, 2006; Straus & Gelles, 1986), a substantial proportion of those experiencing it being injured (Khan & Cooke, 2013; Reese-Weber, 2008), and a range of weapons being used to threaten or attack siblings, including knives, broken glass, and guns (Kiselica & Morrill-Richards, 2007), this behavior has received much less research attention than other forms of familial aggression (DeKeseredy & Ellis, 1997; Wiehe, 1997). This is surprising given that weapon use against siblings has been reported not only in clinical and forensic populations who might be deemed at higher risk for violence (Kuay et al., 2016; Tompsett, Mahoney, & Lackey, 2018) but also in lower-risk community and student populations (Khan, 2017) and extreme sibling aggression also occurs (Salmon & Hehman, 2014).

Archer (2013) noted that while most studies of sibling aggression do not measure the genetic relatedness of perpetrator and victim, living with genetically unrelated brothers and sisters (i.e., stepsiblings) is a robust predictor of intentional and severe sibling aggression perpetration, including the use of weapons (Khan & Cooke, 2008). Parents report less frequent conflict between full siblings than those who are not fully related (Aquilino, 1991) and conflict between non-biological siblings is most intense (Salmon & Hehman, 2015). Furthermore, children living in households with both full and half siblings are at greater risk of injury than those living with full siblings only (Tanskanen, Danielsbacka, & Rotkirch, 2015) although conflict is less intense among half siblings than full siblings (Salmon & Hehman, 2015). The invisibility of sibling aggression in official crime databases or statistics has impinged on efforts to test this hypothesis using violence and homicide data. In a notable exception, Michalski, Russell, Shackelford, and Weekes-Shackelford (2007) examined historical records to explore the influence of genetic relatedness on the nature of aggression used in siblicides. Although they found a relationship between more brutal methods of homicide (e.g., beatings) and genetic relatedness of siblings, it was not statistically significant, and they concluded that there was insufficient reliable data available to examine this effect with full confidence.

The current studies investigated closeness and aggression among full and half siblings. Study 1 examined emotional closeness and conflict, and Study 2 addressed the intentional use of aggression. For both studies, retrospective accounts of relationships with full and half sibling were gathered using self-report questionnaires. If Hamilton’s Rule is operating without any other considerations, we would expect that as the coefficient of relatedness decreased, emotional closeness would decline, while conflict and the use of physical aggression would be more frequent and/or more severe. Specifically, we predicted greater emotional closeness and lower levels of conflict and intentional aggression within relationships with full (*r* = .5) than with half (*r* = .25) siblings.

## Study 1

### Method

#### Participants

Men and women (*N* = 243, 201 female) aged 16 to 21 years (*M* = 17.30, *SD* = .83) were recruited from a British College of Further Education. Questionnaires were completed offline at the college campus. Participation was voluntary and all participants provided informed consent.

#### Materials

Participants completed a questionnaire specifically designed for the study. It contained initial demographic questions, e.g., age and sex, and the age, sex, and type of sibling (full, half, or unrelated). These were followed by 15 statements relating to help and support, lending money and possessions, conflict, and relationship quality which were developed to assess sibling relationship closeness. The items are shown in Table 1. Three of these items concerned conflict (items 2, 4, and 6 in Table 1), and these were analysed separately to provide a measure of conflict. Participants responded to each item on a 5-point scale (1 = “not true at all”, 2 = “not really true”, 3 = “occasionally true”, 4 = “fairly true”, 5 = “very true”). Seven items were worded in a positive direction, seven in a negative direction and one measured perceived similarity to the sibling. The overall sibling relationship quality scale demonstrated acceptable reliability (α = .88). High scores indicated greater levels of sibling closeness. The sibling conflict sub-scale also demonstrated acceptable reliability (α = .84); higher scores indicated greater sibling conflict.

### Results

Participants reported having 462 siblings aged 1 to 39 years (*M* = 17.80, *SD* = 7.09). Of these, 234 were male and 228 female; 349 were full siblings, 75 were half siblings, and 38 were unrelated siblings. Only 23 participants reported having an unrelated sibling (adopted sibling or stepsibling) and 3 of these participants reported having only unrelated siblings.

Due to the few cases of unrelated siblings in the present study, we decided to omit all reports of unrelated siblings from the analysis, which thus focused on comparing full and half siblings. Table 1 shows the frequencies reported for each of the 15 items in the sibling relationship closeness scale; on average emotional closeness scores were lower for full (*M* = 47.86, *SD* = 10.37) than for half (*M* = 52.93, *SD* = 9.77) siblings.

**Table 1:** Percentages of Reports in Study 1 of Different Frequencies of Each of 15 Questions Designed to Assess Perceived Closeness of Relationship to Full Siblings (n = 349) or Half Siblings (n = 75)

Closeness / Sibling type	Not true at all	Not really true	Occasionally true	Fairly true	Very true
1. I get on well with this brother/sister					
Full	3%	5%	25%	32%	34%
Half	4%	4%	11%	38%	45%
2. I argue with this brother/sister (R)^a^					
Full	12%	22%	32%	14%	20%
Half	9%	11%	21%	21%	38%
3. I would lend money to this brother/sister					
Full	14%	14%	24%	14%	36%
Half	5%	7%	9%	29%	50%
4. This brother/sister gets on my nerves (R)^a^					
Full	22%	15%	31%	15%	17%
Half	9%	14%	21%	25%	30%
5. I would happily lend my possessions to this brother/sister					
Full	14%	17%	19%	29%	22%
Half	11%	14%	25%	16%	34%
6. I sometimes get angry and shout at this brother/sister (R)^a^					
Full	25%	12%	41%	14%	89%
Half	13%	20%	13%	25%	30%
7. I feel I am in many ways similar to this brother/sister					
Full	24%	19%	17%	27%	14%
Half	14%	16%	20%	25%	23%
8. I would not willingly lend my possessions to this brother/sister (R)					
Full	19%	9%	17%	15%	41%
Half	7%	9%	11%	25%	48%
9. This person would help me in a time of difficulty					
Full	3%	10%	15%	22%	49%
Half	4%	9%	13%	30%	44%
10. I do not miss this brother/sister when they are away (for example on holiday) (R)					
Full	17%	19%	17%	15%	32%
Half	16%	21%	13%	18%	32%
11. When I am upset I would go to this brother/sister for advice					
Full	24%	17%	20%	9%	31%
Half	27%	23%	16%	16%	18%
12. I would not willingly lend money to this brother/sister (R)					
Full	9%	7%	25%	19%	41%
Half	5%	7%	14%	27%	46%
13. When I am upset I would go to this brother/sister for comfort					
Full	25%	12%	24%	12%	27%
Half	27%	14%	14%	18%	27%
14. I would be happy to help this brother/sister if they were in a difficult situation					
Full	2%	2%	3%	24%	70%
Half	4%	2%	7%	21%	66%
15. I don’t get on very well with this brother/sister (R)					
Full	0	3%	19%	37%	41%
Half	2%	5%	7%	32%	54%

*Note.* Percentages may not total 100% as all figures are rounded; **(R)** indicates a reverse-scored item.

^a^Item denotes act of conflict.

One of the central assumptions underlying Analyses-of-Variance is that all data points are independent. Thus, where participants had more than one sibling and therefore contributed more than one response to the data set, responses were averaged across siblings for each participant. For participants with a half sibling, closeness scores were averaged for all half siblings and their closeness scores for any full siblings were removed from the data set. For the remaining participants (who had only full siblings), closeness scores were averaged for all siblings. This resulted in 194 mean closeness scores for full siblings and 46 mean closeness scores for half siblings. There were no differences between male and female participants in mean closeness scores, *t*(238) = .96, *p* = .34. The differences in age between the respondent and each sibling that contributed to the data were also averaged to give a mean age difference for each participant, both full siblings (*M* = -.11, *SD* = 6.03) and half siblings (*M* = -2.02, *SD* = 10.56). Mean age difference was significantly correlated with mean closeness, *r* = .205, *p* = .001, indicating that participants reported being closer to siblings with whom there was a larger age difference, and that competition between similar age siblings is important. Furthermore, there was a positive correlation between age difference and emotional closeness, *r* = .28, *p* = .001, for participants older than their siblings. This suggested that these participants reported feeling closer to their younger siblings if there was a larger age difference, but not siblings similar to their own age. On the other hand, there was a negative correlation between and emotional closeness, *r* = -.30, *p* = .001, when the participant was the younger sibling, indicating that these participants felt less close to siblings with a larger age gap. We therefore used mean age difference as a covariate in subsequent analyses of the effect of genetic relatedness on closeness scores.

Mean closeness scores for full siblings and half siblings were entered into a general linear model univariate ANCOVA. After controlling for mean age difference, *F*(1, 237) = 10.01, *p* = .002, there was no effect of relatedness group on closeness, *F*(1, 237) = .22, *p* = .642, partial η^2^ = .001, indicating that participants did not report being closer to full siblings than half siblings. We then examined separately the three items in the closeness scale that addressed conflict (i.e., items 2, 4, and 6; see Table 1). In this case, the items were not reversed so that the sum of the items formed a conflict score with higher scores indicating greater conflict. There were no differences between male and female participants in mean conflict scores, *t*(238) = .88, *p* = .38. Age difference significantly correlated with conflict, *r* = .34, *p* < .001, and so mean age difference was entered as a covariate. After controlling for mean age difference, *F*(1, 237) = 13.26, *p* < .001, there was a significant effect of relatedness on conflict, *F*(1, 237) = 5.34, *p* =.022, partial η^2^ = .022, indicating that more conflict (*d* = .43) was reported with full siblings (*M* = 9.27, *SD* = 2.73), than with half siblings (*M* = 8.06, *SD* = 3.11).^i^

Given that the method of averaging across siblings for each participant was a somewhat unorthodox approach, we also adopted a second approach to data analysis which involved the inclusion of all reports of full and half siblings and treating them as independent data. This resulted in 424 reports of sibling relationships (329 full siblings, 75 half siblings) being entered into a univariate ANCOVA with age difference treated as a covariate. Again, after controlling for age difference, *F*(1, 421) = 26.46, *p* < .001, there was no effect of group on closeness scores, *F*(1, 421) = 2.00, *p* = .158, partial η^2^ = .005, but there was a significant effect of group for the three-item conflict score, *F*(1, 421) = 7.68, *p* = .006, indicating that more conflict was reported with full siblings, *M* = 6.61, *SD* = 2.19, than with half siblings, *M* = 5.12, *SD* = 2.59; *d* = .66.

As we did not expect to find more conflict between full siblings than half siblings, and because there were only three items dealing with low level conflict (rather than actual aggressive behavior) on the emotional closeness scale, we conducted a second study that was specifically concerned with sibling aggression.

## Study 2

### Method

#### Participants

Men and women (*N* = 218, 163 female) aged 16 to 55 years (*M* = 23.50, *SD* = 7.16) were recruited from a British College of Further Education and University. Questionnaires were completed offline at the college or university campus. Participation was voluntary and all participants provided informed consent.

#### Materials

Participants completed a questionnaire, which requested the following: (1) demographic information (i.e., age and sex, number of siblings, and type of relationship (full, half, or unrelated) with each sibling). (2) For each sibling, participants responded to 13 items based on those used by Straus et al. (1980) for their US national representative study of different forms of family violence. The items, shown in Table 2, measured the frequency of various intentional acts of aggression against a sibling.

Participants were asked not to include any incidents which were accidental or the result of play-fighting. Frequencies were rated along a 6-point scale (0 = “never”, 1 = “very rarely”, 2 = “rarely”, 3 = “sometimes”, 4 = “often”, 5 = “very often”). The overall sibling conflict measure demonstrated acceptable reliability (α = .87). High scores indicated greater levels of sibling conflict.

### Results

Participants reported 422 sibling relationships, including 317 full, 81 half, and 24 unrelated siblings. Table 2 shows the frequencies reported for each of the 13 items in the intentional aggression scale, for full and half siblings.

**Table 2:** Percentages of Reports in Study 2 of Different Frequencies of Each of 13 Acts of Aggression Towards Full Siblings (n = 317) or Half Siblings (n = 81)

Act of aggression / Sibling type	Never	Very rarely	Rarely	Sometimes	Often	Very often
1. Kicked or bitten						
Full	44%	11%	21%	17%	5%	2%
Half	68%	6%	10%	14%	3%	0
2. Punched						
Full	44%	13%	17%	16%	8%	2%
Half	69%	6%	6%	12%	6%	0
3. Threw heavy/sharp object^a^						
Full	59%	10%	16%	10%	4%	1%
Half	78%	7%	6%	7%	1%	0
4. Slapped						
Full	45%	15%	18%	12%	7%	3%
Half	67%	10%	12%	9%	1%	1%
5. Beaten						
Full	77%	6%	9%	4%	3%	1%
Half	91%	1%	1%	4%	3%	0
6. Attempted to strangle						
Full	92%	4%	3%	1%	1	0
Half	96%	1%	0	1%	1%	0
7. Threatened with knife^a^						
Full	95%	3%	0	1%	0	0
Half	96%	4%	0	0	0	0
8. Used a knife^a^						
Full	100%	0	0	0	0	0
Half	100%	0	0	0	0	0
9. Pushed						
Full	36%	9%	22%	22%	9%	3%
Half	54%	9%	24%	10%	4%	0
10. Threatened with gun^a^						
Full	100%	0	0	0	0	0
Half	99%	0	0	0	1%	0
11. Grabbed						
Full	48%	10%	19%	17%	5%	1%
Half	69%	7%	12%	7%	4%	0
12. Used a gun^a^						
Full	100%	0	0	0	0	0
Half	100%	0	0	0	0	0
13. Other serious act^a^						
Full	98%	1%	0	0	0	0
Half	97%	3%	1%	0	0	0

*Note.* Percentages may not total 100% as all figures are rounded.

^a^Item denotes aggressive acts with the use or threat of weapons.

Following the same procedure used to recode the data from Study 1, relationships involving unrelated siblings were removed from the dataset, responses from each participant with half siblings (*n* = 42) were averaged across all half siblings (and their full sibling relationships removed), and responses from each participant with only full siblings (*n* = 172) were averaged across all full siblings. An independent samples t-test showed that there were no sex differences in the overall level of aggression reported, *t*(212) = .06, *p* = .955, and so participant sex played no further part in the analyses. Participants reported significantly more frequent physically aggressive behavior overall towards full siblings (*M* = 8.92, *SD* = 7.34) than towards half siblings (*M* = 4.42, *SD* = 5.58), *t*(212) = 3.71, *p* < .001, *d* = .64.

Following the analysis in Study 1, we also took a second approach to data analysis that treated all responses as independent data rather than averaging across responses for each participant. The resulting analysis comparing all responses involving a full sibling (*n* = 317) with all responses involving a half sibling (*n* = 81) was consistent with the main analysis. Participants reported more frequent aggressive behavior overall towards full siblings (*M* = 8.61, *SD* = 8.09) than towards half siblings (*M* = 4.93, *SD* = 7.36), *t*(396) = 2.39, *p* = .017 (*d* = .46).

## Discussion

The present study investigated closeness and frequency of physically aggressive behavior in full and half sibling relationships. Contrary to Hamilton’s Rule (Hamilton, 1964a, 1964b), we found no effect of relatedness in Study 1 for closeness when age differences were controlled. In both studies, we found higher levels of conflict or aggression between full siblings than between half siblings.

To consider these findings within an evolutionary framework, we note that according to “Hamilton’s rule” (Buss, 1999, 2011), altruistic behavior will only occur when the benefits from helping relatives exceed the costs of doing so. These cost-benefit considerations can explain both altruistic behavior and aggression between close relatives, the latter reflecting competition for resources. Although full, half, and unrelated siblings typically receive parental investment, parents favor biological over non-biological children (e.g., Daly & Wilson, 1994, 2008). Hence, full siblings receive a greater proportion of parental investment and constitute a greater threat to the acquisition of valued resources. These resources have a substantial impact on sibling wellbeing (e.g., physical development: Lawson & Mace, 2008; education: Steelman et al., 2002; and health: Zeng et al., 2013). These findings are consistent with the suggestion that there is diluted resource competition over parental investment between half-siblings in societies with serial monogamy as sources of parental investment are only partly overlapping among half-siblings (Tanskanen, Danielsbacka, Jokela, David-Barrett, & Rotkirch, 2016).

A number of variables are predicted to increase the intensity of this competition and future research should consider the relative influence of these. For example, the level of household resources available to parents influences the allocation of parental investment (Beaulieu & Bugental, 2008). In non-human species, sibling aggression is greater when resources are limited (e.g., Drummond & Garcia Chavelas, 1989; Mock & Parker, 1997), and in humans, a severe lack of resources may result in investment in one child only. However, an abundance of parental resources does not necessarily reduce the frequency or severity of sibling competition and greater access to resources may actually increase motivation for sibling-oriented aggression. Consistent with this, Gibson and Lawson (2011) demonstrated that modernization and subsequent greater access to resources are associated with greater sibling competition. Additional research is required to investigate the importance of resource availability, differential allocation of resources between siblings, and the extent to which perceived parental favoritism (Goodwin & Roscoe, 1990; Hoffman & Edwards, 2004; Roscoe, Goodwin, & Kennedy, 1987) impacts on this association. As the extent to which competition is ‘local’ may influence the frequency and level of cooperative and competitive behavior (West et al., 2006) future research may consider the extent to which siblings shared resources (e.g., shared bedroom).

The structure of the family unit (e.g., child birth order) is also likely to influence sibling dynamics. For example, the number of siblings may be detrimental to later-born children (Lawson & Mace, 2009), while young children can benefit from resources generated by older siblings (Sawada & Lokshin, 2009). Closeness and aggression may further differ between maternal and paternal half siblings. Due to paternity uncertainty and the prevalence of cuckoldry (Platek & Shackelford, 2006), a minority of paternal half siblings will be unrelated. Overall, genetic relatedness is higher in matrilineal than in patrilineal kin (Michalski & Euler, 2008) and more frequent contact has been identified between maternal than paternal half siblings, although generational differences exist (Tanskanen & Danielsbacka, 2014). Future research would benefit from comparing relationships with maternal and paternal half siblings, and the extent to which these differ from relationships with full siblings.

The present study investigated full and half siblings. Future studies may extend these findings and compare relationships with categories of unrelated siblings, i.e., adopted and stepsiblings. While adopted and stepsiblings are both genetically unrelated (and therefore fall into the same category of relatedness), their status is very different. For example, the circumstances that lead to children living with an adoptive parent or a stepparent are likely to be markedly different (Kreider & Lofquist, ‎2010). Thus, an adopted child who joins a family in which they form part of a sibling-set is more actively ‘wanted’ or ‘desired’ than stepsiblings. Consistent with this, there is greater maternal involvement in adoptive families (Rhea & Corley, 1994), and adoptive families invest greater resources (Hamilton, Cheng, & Powell, 2007) particularly in the education (Gibson, 2009) and health of the child (Bramlett, Radel, & Blumberg, 2007).

The extent of conflict between siblings is likely to be related to the amount of time that they spent living in the same residence (and thus, covary with the extent of competition for resources), although the number of cohabitation years is itself not a perfect measure of the extent of competition. In order to minimize the impact of variation in cohabitation years, Study 2 asked respondents to report on the frequency of aggression rather than estimating the number of acts of aggression, using the rationale that the frequency of behavior transcends the time period over which that behavior occurs. Nevertheless, the inclusion of cohabitation years alongside frequency estimates of aggression would allow a more comprehensive analysis of sibling conflict and future investigations should consider this approach.

While sex differences in aggression are widely reported (Archer, 2004, 2009; Card, Stucky, Sawalani, & Little, 2008), no sex differences were apparent in our data with respect to reported levels of conflict (Study 1) or aggression (Study 2). While our findings do not support the sex differences in sibling conflict found in some studies (e.g., Campione‐Barr & Smetana, 2010; Salmon & Hehman, 2015; White & Riedmann, 1992), they corroborate research that reports no difference between male and female siblings’ use of aggression (e.g., Felson, 1983; Minnett, Vandell, & Santrock, 1983; Roscoe et al., 1987; Stock, 1993). One explanation for our findings might be that the data from Study 1 were reports of the frequency of low-level conflict (e.g., irritation, arguing) where the expectation of sex differences may be much less clear. It might also be that expectations of sex differences in aggressive behavior (i.e., more male than female aggression) are driven by observations of conflict interactions between strangers or acquaintances whereas a rather different picture emerges from observations of partner conflict and parental conflict. In these latter familial and partner relationships, it is not uncommon to find more female aggression than male aggression (Archer, 2000; Bates, Graham-Kevan, & Archer, 2014; Straus & Gelles, 1986; Straus et al., 1980).^ii^

The diversity in sibling structures raises several measurement issues that can only be answered by subsequent studies that collect a wider range of background variables from participants. There are, for example, mixed results for the influence of sibling-dyad’s sex on aggression (see Goodwin & Roscoe, 1990; Hoffman & Edwards, 2004; Pepler, Abramovitch, & Corter, 1981). However, Salmon and Hehman (2015) found this to be pertinent in relation to sibling conflict. Though respondent sex did not predict the intensity of sibling conflict, more intense conflict occurred between siblings of the same than opposite sex. Same-sex siblings are more likely to share bedrooms or spend time together on sports-teams or in social-clubs; hence there are more opportunities to compete for resources, peers, or mates. Thus, for a fuller examination, future studies would benefit from measuring the influence of same/opposite sex of sibling as a contributory factor. Longitudinal research is also recommended to explore these issues as sibling relationships change over time (Pollet & Hoben, 2011). Future studies might also benefit from more specific measures of sibling closeness that separate this from related concepts such as altruism. A single-item measure of emotional closeness may also be useful for comparison purposes (i.e., asking respondents “how close to you feel to this sibling?”).

Assessments of physical aggression were collected using self-reported ratings of the frequency of a range of violent behavior used against each sibling. It is noteworthy that these self-report data may have been influenced by social desirability and the ‘softening’ effect of memory bias (Wilson & Fromuth, 1997), possibly as a result of the normalization of sibling aggression during childhood (Khan & Rogers, 2015). Nevertheless, there is a widespread history of using anonymous self-reports to assess aggression in adults where other methods used for children, such as observations, and peer or teacher reports, are not appropriate. Finally, despite varying in grievousness, each form of behavior was equally weighted to aggregate an index of aggression; this is because we wished to record the frequency of aggression rather than the acuteness or intention to harm. Nevertheless, aggression towards others may be indexed in terms of severity as well as frequency, and these individual facets of aggression may be independent of each other and not necessarily correlated. Thus, future work could also assess how sibling relatedness impacts on the severity of physical aggression as well as its frequency. Research addressing a range of covert and overt physical and non-physical behaviors would be beneficial, together with further investigation of prosocial cooperative interactions.

The current study was reliant on a British student sample, consistent with the dominance of ‘WEIRD’ participants (Western, Educated, from Industrialised, Rich, Democratic countries) in psychological research (Henrich, Heine, & Norenzayan, 2010). Previous research has demonstrated cultural differences in cooperative and competitive sibling behavior which does not appear to be a consequence of differences in family structure (such as number of siblings) between cultural groups (Knight, Kagan, & Knight, 1982). Culture may also influence parental treatment of siblings and equality of sibling treatment (McHale, Updegraff, Shanahan, Crouter, & Killoren, 2005). Future research should adopt a wider cross-cultural sample and consider factors such as the customs of child rearing and the environment in which the child lives (Super & Harkness, 1986). Comparisons of individualistic and collectivistic cultures would be of particular interest (Buist et al., 2014; Oetzel et al., 2003). Though previous research suggests that ethnicity does not impact on family conflict (e.g., Formoso, Gonzales, & Aiken, 2000), studies recruiting from one country may also explore the importance of this demographic variable.

To conclude, the present studies indicate that relationships with full siblings involve levels of emotional closeness that are no different to those with half siblings and that they involve higher levels of aggression than in relationships with half siblings. While apparently inconsistent with Hamilton’s rule (Hamilton, 1964a, 1964b), these findings may reflect the other side of this rule, the extent to which siblings constitute rivals for parental investment, which is disproportionately provided to biological offspring. We encourage further research that explores not only the influence of genetic relatedness on sibling aggression, but also their competition for resources, not least because blended sibling relationships are socially ubiquitous yet often overlooked by family aggression researchers.
